# 2-[(2-Amino­phen­yl)sulfan­yl]-*N*-(4-meth­oxy­phen­yl)acetamide

**DOI:** 10.1107/S1600536812024178

**Published:** 2012-05-31

**Authors:** Shahzad Murtaza, M. Nawaz Tahir, Javaria Tariq, Aadil Abbas, Naghmana Kausar

**Affiliations:** a Department of Chemistry, University of Gujrat, Hafiz Hayat Campus, Gujrat, Pakistan; b Department of Physics, University of Sargodha, Sargodha, Pakistan

## Abstract

In the title compound, C_15_H_16_N_2_O_2_S, the dihedral angle between the 4-meth­oxy­aniline and 2-amino­benzene­thiole fragments is 35.60 (9)°. A short intra­molecular N—H⋯S contact leads to an *S*(5) ring. In the crystal, mol­ecules are consolidated in the form of polymeric chains along [010] as a result of N—H⋯O hydrogen bonds, which generate *R*_3_^2^(18) and *R*_4_^3^(22) loops. The polymeric chains are interlinked through C—H⋯O inter­action and complete *R*_2_^2^(8) ring motifs.

## Related literature

For a related structure, see: Haisa *et al.* (1980 ▶). For hydrogen-bond motif notation, see: Bernstein *et al.* (1995 ▶). 
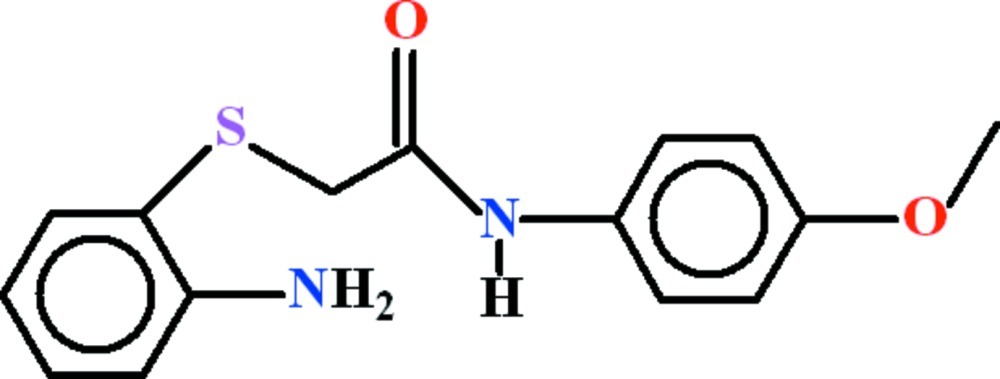


## Experimental

### 

#### Crystal data

C_15_H_16_N_2_O_2_S
*M* *_r_* = 288.36
Monoclinic, 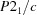

*a* = 12.9935 (16) Å
*b* = 4.7990 (4) Å
*c* = 23.433 (3) Å
β = 95.506 (7)°
*V* = 1454.4 (3) Å^3^
*Z* = 4
Mo *K*α radiation
μ = 0.23 mm^−1^
*T* = 296 K
0.25 × 0.14 × 0.12 mm

#### Data collection

Bruker Kappa APEXII CCD diffractometer
Absorption correction: multi-scan (*SADABS*; Bruker, 2005 ▶) *T*_min_ = 0.965, *T*_max_ = 0.975
11532 measured reflections
2845 independent reflections
1525 reflections with *I* > 2σ(*I*)
*R*_int_ = 0.060

#### Refinement

*R*[*F* ^2^ > 2σ(*F* ^2^)] = 0.050
*wR*(*F* ^2^) = 0.121
*S* = 1.01
2845 reflections
182 parameters
H-atom parameters constrained
Δρ_max_ = 0.18 e Å^−3^
Δρ_min_ = −0.20 e Å^−3^

### 

Data collection: *APEX2* (Bruker, 2009 ▶); cell refinement: *SAINT* (Bruker, 2009 ▶); data reduction: *SAINT*; program(s) used to solve structure: *SHELXS97* (Sheldrick, 2008 ▶); program(s) used to refine structure: *SHELXL97* (Sheldrick, 2008 ▶); molecular graphics: *ORTEP-3 for Windows* (Farrugia, 1997 ▶) and *PLATON* (Spek, 2009 ▶); software used to prepare material for publication: *WinGX* (Farrugia, 1999 ▶) and *PLATON*.

## Supplementary Material

Crystal structure: contains datablock(s) global, I. DOI: 10.1107/S1600536812024178/hb6821sup1.cif

Structure factors: contains datablock(s) I. DOI: 10.1107/S1600536812024178/hb6821Isup2.hkl

Supplementary material file. DOI: 10.1107/S1600536812024178/hb6821Isup3.cml

Additional supplementary materials:  crystallographic information; 3D view; checkCIF report

## Figures and Tables

**Table 1:** Hydrogen-bond geometry (Å, °)

*D*—H⋯*A*	*D*—H	H⋯*A*	*D*⋯*A*	*D*—H⋯*A*
N2—H2*B*⋯S1	0.86	2.60	3.004 (3)	110
N1—H1⋯O2^i^	0.86	2.00	2.848 (3)	170
N2—H2*A*⋯O2^ii^	0.86	2.38	3.200 (3)	161
C3—H3⋯O1^iii^	0.93	2.47	3.393 (5)	170

Symmetry codes: (i) 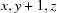; (ii) 
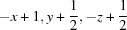
; (iii) 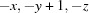.

**Fig. 1.**
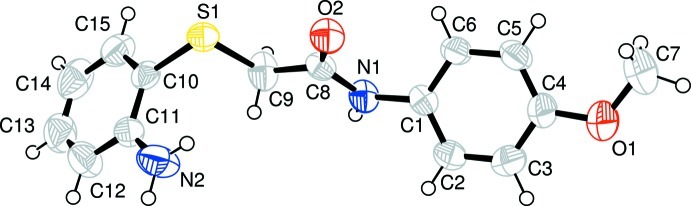
View of the title compound with displacement ellipsoids drawn at the 50% probability level.

**Fig. 2.**
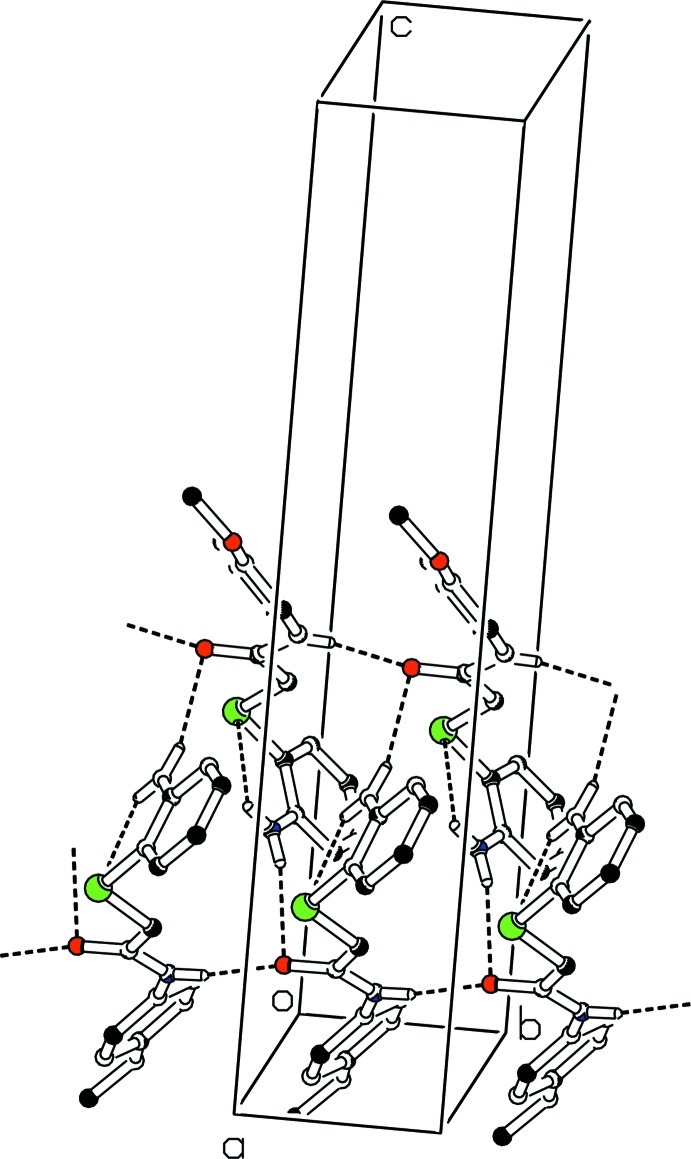
The partial packing, which shows that molecules form polymeric chains with various ring motifs. The H-atoms not involved in H-bondings are omitted for clarity.

## supplementary crystallographic information

### Comment

The title compound (I), (Fig. 1) has been synthesized to check its biological application as antimicrobial agent owing to the concept that amide moiety is an important part of different drugs.

The crystal structure of *N*-(4-methoxyphenyl)acetamide (Haisa *et al.*, 1980) has been published which is related to the title compound (I, Fig. 1).

In (I), the 4-methoxyanilinic and 2-aminobenzenethiolic groups A (C1–C7/N1/O1) and B (C10—C15/N2/S1) are almost planar with r. m. s. deviation of 0.0150 Å and 0.0134 Å, respectively. The dihedral angle between A/B is 35.60 (9)°. The central acetamide moiety C (C8/C9/O2) is of course planar. The dihedral angle between A/C and B/C is 48.43 (10)° and 78.07 (8)°, respectively. There exist S(5) ring motif (Bernstein *et al.*, 1995) due to H-bonding of N—H···S type (Table 1, Fig. 2). The molecules are stabilized in the form of one-dimensional polymeric network due to H-bondings (Table 1, Fig. 2) of N—H···O type. There exist *R*_3_^2^(18) and *R*_4_^3^(22) ring motifs in the polymeric network when three and four molecules respectively, are connected with each other.

### Experimental

2-Aminobenzothiol (0.125 g, 0.5 mmol) was dissolved in anhydrous diethylether (10 ml) and NaH (0.024 g, 1 mmol) was added to it at temperature 273–278 K. A separately prepared solution of 2-chloro-*N*-(4-methoxyphenyl)acetamide (0.1 g, 0.5 mmol) in anhydrous diethylether (10 ml) was added drop wise to above mixture. The mixture was stirred for 4 h and solvent was evaporated to get greyish crystals of (I). m.p. 400 K

### Refinement

The H-atoms were positioned geometrically (N—H = 0.86, C–H = 0.93–0.97 Å) and were included in the refinement in the riding model approximation, with *U*_iso_(H) = *xU*_eq_(C, N), where *x* = 1.5 for CH_3_ and *x* = 1.2 for other H-atoms.

### Crystal data

**Table:**

C_15_H_16_N_2_O_2_S	*F*(000) = 608
*M**_r_* = 288.36	*D*_x_ = 1.317 Mg m^−^^3^
Monoclinic, *P*2_1_/*c*	Mo *K*α radiation, λ = 0.71073 Å
Hall symbol: -P 2ybc	Cell parameters from 1525 reflections
*a* = 12.9935 (16) Å	θ = 1.6–26.0°
*b* = 4.7990 (4) Å	µ = 0.23 mm^−^^1^
*c* = 23.433 (3) Å	*T* = 296 K
β = 95.506 (7)°	Needle, gray
*V* = 1454.4 (3) Å^3^	0.25 × 0.14 × 0.12 mm
*Z* = 4	

### Data collection

**Table:**

Bruker Kappa APEXII CCD diffractometer	2845 independent reflections
Radiation source: fine-focus sealed tube	1525 reflections with *I* > 2σ(*I*)
Graphite monochromator	*R*_int_ = 0.060
Detector resolution: 7.80 pixels mm^-1^	θ_max_ = 26.0°, θ_min_ = 1.6°
ω scans	*h* = −16→16
Absorption correction: multi-scan (*SADABS*; Bruker, 2005)	*k* = −5→5
*T*_min_ = 0.965, *T*_max_ = 0.975	*l* = −27→28
11532 measured reflections	

### Refinement

**Table:**

Refinement on *F*^2^	Primary atom site location: structure-invariant direct methods
Least-squares matrix: full	Secondary atom site location: difference Fourier map
*R*[*F*^2^ > 2σ(*F*^2^)] = 0.050	Hydrogen site location: inferred from neighbouring sites
*wR*(*F*^2^) = 0.121	H-atom parameters constrained
*S* = 1.01	*w* = 1/[σ^2^(*F*_o_^2^) + (0.0435*P*)^2^ + 0.1361*P*] where *P* = (*F*_o_^2^ + 2*F*_c_^2^)/3
2845 reflections	(Δ/σ)_max_ < 0.001
182 parameters	Δρ_max_ = 0.18 e Å^−^^3^
0 restraints	Δρ_min_ = −0.20 e Å^−^^3^

### Special details

**Table:**

Geometry. Bond distances, angles *etc*. have been calculated using the rounded fractional coordinates. All su's are estimated from the variances of the (full) variance-covariance matrix. The cell e.s.d.'s are taken into account in the estimation of distances, angles and torsion angles
Refinement. Refinement of *F*^2^ against ALL reflections. The weighted *R*-factor *wR* and goodness of fit *S* are based on *F*^2^, conventional *R*-factors *R* are based on *F*, with *F* set to zero for negative *F*^2^. The threshold expression of *F*^2^ > σ(*F*^2^) is used only for calculating *R*-factors(gt) *etc*. and is not relevant to the choice of reflections for refinement. *R*-factors based on *F*^2^ are statistically about twice as large as those based on *F*, and *R*- factors based on ALL data will be even larger.

### Fractional atomic coordinates and isotropic or equivalent isotropic displacement parameters (Å^2^)

**Table:**

	*x*	*y*	*z*	*U*_iso_*/*U*_eq_	
S1	0.67008 (6)	0.17005 (14)	0.17482 (3)	0.0513 (3)	
O1	0.06467 (19)	0.2484 (5)	−0.05402 (11)	0.0942 (11)	
O2	0.48924 (15)	0.0421 (3)	0.09749 (9)	0.0565 (8)	
N1	0.43663 (19)	0.4757 (4)	0.07298 (10)	0.0480 (9)	
N2	0.5848 (2)	0.4083 (5)	0.27906 (12)	0.0763 (12)	
C1	0.3429 (2)	0.4077 (5)	0.03984 (12)	0.0423 (10)	
C2	0.2538 (3)	0.5466 (6)	0.04982 (14)	0.0578 (11)	
C3	0.1626 (3)	0.4868 (7)	0.01822 (16)	0.0750 (14)	
C4	0.1593 (3)	0.2887 (7)	−0.02452 (14)	0.0590 (12)	
C5	0.2468 (3)	0.1500 (6)	−0.03442 (13)	0.0569 (11)	
C6	0.3386 (2)	0.2099 (6)	−0.00259 (12)	0.0496 (11)	
C7	0.0533 (3)	0.0393 (9)	−0.09623 (17)	0.1020 (17)	
C8	0.5023 (2)	0.2953 (5)	0.09956 (11)	0.0423 (10)	
C9	0.5937 (2)	0.4227 (5)	0.13308 (13)	0.0606 (11)	
C10	0.7332 (2)	0.4001 (5)	0.22519 (13)	0.0467 (11)	
C11	0.6839 (3)	0.4890 (6)	0.27227 (14)	0.0520 (11)	
C12	0.7355 (3)	0.6709 (7)	0.31078 (15)	0.0743 (14)	
C13	0.8316 (3)	0.7639 (7)	0.30315 (18)	0.0838 (17)	
C14	0.8816 (3)	0.6776 (7)	0.25769 (19)	0.0842 (16)	
C15	0.8321 (3)	0.4935 (7)	0.21861 (16)	0.0654 (14)	
H1	0.45235	0.64944	0.07614	0.0576*	
H2	0.25569	0.68219	0.07826	0.0696*	
H2A	0.55421	0.47092	0.30741	0.0916*	
H2B	0.55300	0.29502	0.25497	0.0916*	
H3	0.10255	0.58008	0.02558	0.0897*	
H5	0.24472	0.01413	−0.06280	0.0680*	
H6	0.39837	0.11528	−0.00991	0.0595*	
H7A	0.07153	−0.13793	−0.07913	0.1529*	
H7B	0.09784	0.07889	−0.12562	0.1529*	
H7C	−0.01719	0.03419	−0.11275	0.1529*	
H9A	0.57022	0.56384	0.15846	0.0724*	
H9B	0.63654	0.51361	0.10692	0.0724*	
H12	0.70365	0.73062	0.34250	0.0890*	
H13	0.86413	0.88868	0.32937	0.1003*	
H14	0.94784	0.74134	0.25299	0.1009*	
H15	0.86569	0.43201	0.18764	0.0783*	

### Atomic displacement parameters (Å^2^)

**Table:**

	*U*^11^	*U*^22^	*U*^33^	*U*^12^	*U*^13^	*U*^23^
S1	0.0592 (5)	0.0369 (4)	0.0572 (5)	0.0064 (4)	0.0021 (4)	−0.0061 (4)
O1	0.0605 (17)	0.114 (2)	0.103 (2)	0.0024 (14)	−0.0178 (16)	−0.0410 (17)
O2	0.0715 (15)	0.0257 (10)	0.0700 (15)	−0.0040 (9)	−0.0054 (12)	−0.0028 (10)
N1	0.0592 (17)	0.0250 (12)	0.0578 (17)	−0.0080 (11)	−0.0050 (14)	−0.0015 (11)
N2	0.073 (2)	0.086 (2)	0.074 (2)	−0.0108 (16)	0.0276 (17)	−0.0247 (16)
C1	0.054 (2)	0.0314 (15)	0.0412 (18)	−0.0073 (14)	0.0034 (15)	0.0019 (13)
C2	0.065 (2)	0.0463 (18)	0.061 (2)	0.0043 (17)	0.0005 (19)	−0.0163 (16)
C3	0.056 (2)	0.080 (2)	0.088 (3)	0.0132 (19)	0.002 (2)	−0.027 (2)
C4	0.050 (2)	0.062 (2)	0.063 (2)	−0.0052 (17)	−0.0051 (18)	−0.0093 (18)
C5	0.062 (2)	0.0558 (19)	0.053 (2)	−0.0040 (17)	0.0063 (18)	−0.0160 (17)
C6	0.0485 (19)	0.0511 (17)	0.0498 (19)	−0.0019 (15)	0.0082 (16)	−0.0111 (16)
C7	0.091 (3)	0.112 (3)	0.097 (3)	−0.015 (3)	−0.022 (2)	−0.035 (3)
C8	0.055 (2)	0.0301 (15)	0.0423 (18)	−0.0035 (14)	0.0069 (15)	−0.0016 (14)
C9	0.073 (2)	0.0386 (17)	0.066 (2)	−0.0118 (15)	−0.0146 (19)	0.0056 (16)
C10	0.0467 (19)	0.0360 (16)	0.056 (2)	0.0069 (13)	−0.0020 (16)	−0.0011 (14)
C11	0.058 (2)	0.0446 (17)	0.052 (2)	0.0073 (16)	−0.0016 (18)	−0.0048 (16)
C12	0.086 (3)	0.074 (2)	0.061 (2)	−0.003 (2)	−0.002 (2)	−0.017 (2)
C13	0.084 (3)	0.072 (3)	0.089 (3)	0.001 (2)	−0.024 (3)	−0.021 (2)
C14	0.054 (2)	0.072 (2)	0.122 (4)	−0.010 (2)	−0.015 (2)	−0.006 (3)
C15	0.051 (2)	0.064 (2)	0.081 (3)	0.0049 (18)	0.005 (2)	−0.003 (2)

### Geometric parameters (Å, º)

**Table:**

S1—C9	1.796 (3)	C10—C11	1.395 (4)
S1—C10	1.760 (3)	C11—C12	1.382 (5)
O1—C4	1.365 (5)	C12—C13	1.354 (5)
O1—C7	1.407 (5)	C13—C14	1.364 (6)
O2—C8	1.227 (3)	C14—C15	1.386 (5)
N1—C1	1.418 (4)	C2—H2	0.9300
N1—C8	1.328 (3)	C3—H3	0.9300
N2—C11	1.369 (5)	C5—H5	0.9300
N1—H1	0.8600	C6—H6	0.9300
N2—H2A	0.8600	C7—H7A	0.9600
N2—H2B	0.8600	C7—H7B	0.9600
C1—C2	1.375 (4)	C7—H7C	0.9600
C1—C6	1.372 (4)	C9—H9A	0.9700
C2—C3	1.366 (5)	C9—H9B	0.9700
C3—C4	1.379 (5)	C12—H12	0.9300
C4—C5	1.357 (5)	C13—H13	0.9300
C5—C6	1.375 (5)	C14—H14	0.9300
C8—C9	1.491 (4)	C15—H15	0.9300
C10—C15	1.384 (5)		
			
C9—S1—C10	98.01 (12)	C13—C14—C15	119.0 (4)
C4—O1—C7	119.1 (3)	C10—C15—C14	120.7 (3)
C1—N1—C8	125.9 (2)	C1—C2—H2	120.00
C8—N1—H1	117.00	C3—C2—H2	120.00
C1—N1—H1	117.00	C2—C3—H3	120.00
C11—N2—H2A	120.00	C4—C3—H3	120.00
H2A—N2—H2B	120.00	C4—C5—H5	120.00
C11—N2—H2B	120.00	C6—C5—H5	120.00
N1—C1—C6	122.0 (2)	C1—C6—H6	120.00
N1—C1—C2	119.2 (2)	C5—C6—H6	120.00
C2—C1—C6	118.8 (3)	O1—C7—H7A	109.00
C1—C2—C3	120.4 (3)	O1—C7—H7B	109.00
C2—C3—C4	120.2 (3)	O1—C7—H7C	109.00
C3—C4—C5	119.6 (3)	H7A—C7—H7B	109.00
O1—C4—C3	115.4 (3)	H7A—C7—H7C	109.00
O1—C4—C5	124.9 (3)	H7B—C7—H7C	109.00
C4—C5—C6	120.2 (3)	S1—C9—H9A	109.00
C1—C6—C5	120.7 (3)	S1—C9—H9B	109.00
O2—C8—N1	123.2 (2)	C8—C9—H9A	109.00
O2—C8—C9	121.8 (2)	C8—C9—H9B	109.00
N1—C8—C9	115.0 (2)	H9A—C9—H9B	108.00
S1—C9—C8	112.38 (17)	C11—C12—H12	119.00
S1—C10—C15	120.4 (2)	C13—C12—H12	119.00
C11—C10—C15	119.4 (3)	C12—C13—H13	119.00
S1—C10—C11	120.2 (2)	C14—C13—H13	119.00
N2—C11—C12	120.4 (3)	C13—C14—H14	121.00
C10—C11—C12	118.6 (3)	C15—C14—H14	121.00
N2—C11—C10	120.9 (3)	C10—C15—H15	120.00
C11—C12—C13	121.2 (3)	C14—C15—H15	120.00
C12—C13—C14	121.2 (4)		
			
C10—S1—C9—C8	−158.8 (2)	O1—C4—C5—C6	179.1 (3)
C9—S1—C10—C11	82.1 (2)	C3—C4—C5—C6	−1.0 (5)
C9—S1—C10—C15	−98.1 (3)	C4—C5—C6—C1	0.7 (5)
C7—O1—C4—C3	−176.6 (3)	O2—C8—C9—S1	−6.7 (3)
C7—O1—C4—C5	3.3 (5)	N1—C8—C9—S1	172.7 (2)
C8—N1—C1—C2	131.0 (3)	S1—C10—C11—N2	−2.5 (4)
C8—N1—C1—C6	−50.0 (4)	S1—C10—C11—C12	−179.6 (2)
C1—N1—C8—O2	1.2 (4)	C15—C10—C11—N2	177.8 (3)
C1—N1—C8—C9	−178.2 (2)	C15—C10—C11—C12	0.6 (4)
N1—C1—C2—C3	179.5 (3)	S1—C10—C15—C14	179.1 (3)
C6—C1—C2—C3	0.4 (4)	C11—C10—C15—C14	−1.1 (5)
N1—C1—C6—C5	−179.4 (3)	N2—C11—C12—C13	−176.7 (3)
C2—C1—C6—C5	−0.4 (4)	C10—C11—C12—C13	0.5 (5)
C1—C2—C3—C4	−0.8 (5)	C11—C12—C13—C14	−1.1 (6)
C2—C3—C4—O1	−179.0 (3)	C12—C13—C14—C15	0.6 (6)
C2—C3—C4—C5	1.1 (5)	C13—C14—C15—C10	0.5 (5)

### Hydrogen-bond geometry (Å, º)

**Table:**

*D*—H···*A*	*D*—H	H···*A*	*D*···*A*	*D*—H···*A*
N2—H2*B*···S1	0.86	2.60	3.004 (3)	110
N1—H1···O2^i^	0.86	2.00	2.848 (3)	170
N2—H2*A*···O2^ii^	0.86	2.38	3.200 (3)	161
C3—H3···O1^iii^	0.93	2.47	3.393 (5)	170

Symmetry codes: (i) *x*, *y*+1, *z*; (ii) −*x*+1, *y*+1/2, −*z*+1/2; (iii) −*x*, −*y*+1, −*z*.
